# Prevalence of preoperative cognitive impairment among elderly thoracic surgery patients and association with postoperative delirium: a prospective observational study

**DOI:** 10.3389/fnhum.2023.1234018

**Published:** 2023-07-20

**Authors:** Fangfang Li, Mengrong Miao, Ningning Li, Jun Zhou, Mingyang Sun, Jiaqiang Zhang

**Affiliations:** Department of Anesthesiology and Perioperative Medicine, People’s Hospital of Zhengzhou University, Henan Provincial People’s Hospital, Zhengzhou, Henan, China

**Keywords:** preoperative cognitive impairment, Mini-Cog test, postoperative delirium, geriatric patients, thoracic surgery

## Abstract

**Background:**

Preoperative cognitive impairment (PCI) may increase the incidence of postoperative delirium (POD), yet screening for cognitive impairment is rarely performed. This study hypothesized that Mini-Cog for preoperative cognitive impairment screening predicts postoperative delirium.

**Methods:**

The prospective observational study recruited 153 elderly patients presenting for elective thoracic surgery. Cognitive function of these patients was screened using Mini-Cog preoperatively. We considered that patients with Mini-Cog scores ≤ 3 had cognitive impairment. Delirium was assessed using the Short CAM scale on postoperative days 1–5.

**Results:**

Of the 153 participants, 54 (35.3%) were assigned to the PCI group, and 99 (64.7%) were assigned to the Normal group. Place of residence, education level, and history of hypertension were significantly different between the two groups (*P* < 0.05). 51 (33.3%) patients developed POD. Multifactorial analysis revealed that PCI (OR = 2.37, *P* = 0.028), older age (OR = 1.13, *P* = 0.009), ASA grade III (OR = 2.75, *P* = 0.012), and longer duration of anesthesia (OR = 1.01, *P* = 0.007) were associated with POD.

**Conclusion:**

Preoperative cognitive impairment is strongly associated with POD. Mini-Cog could be recommended for screening PCI.

**Clinical trial registration:**

ClinicalTrials.gov, identifier NCT05798767.

## 1. Introduction

In an increasingly aging era, the number of elderly patients requiring surgery continues to grow (Chow et al., 2012). Senile patients have higher rates of perioperative complications and multiple preoperative co-morbidities. Postoperative delirium (POD) is an abnormal mental state with marked fluctuations that occurs suddenly after surgery, which plagues 20–80% of geriatric surgical patients (Inouye, 2006). POD prolongs the patient’s hospital stay, increases medical costs, and increases morbidity and mortality in elderly patients (Robinson et al., 2009; Steinmetz et al., 2009; Goldberg et al., 2020).

Preoperative assessment of risk in major organ systems is routinely performed (Ficarra, 1949). However, cognition assessment has not been included in regular preoperative screening due to limited clinical testing methods (Crosby et al., 2011). Cognitive impairment can be described as a state of different severity of impairment in cognitive function (Petersen, 2004; Langa and Levine, 2014). Epidemiological surveys show that 35–50% of older adults suffer from cognitive impairment (Ward et al., 2012). Recent studies have demonstrated that preoperative cognitive impairment (PCI) relates to POD and leads to other adverse outcomes (Hanna et al., 2020; Chen et al., 2022). The significance of preoperative cognitive assessment should be deeply recognized, which brings many benefits (Pas et al., 2022; Tran et al., 2022).

There is no consensus on the appropriate cognitive assessment tool in the preoperative setting. Being easy to perform clinically and effective should be one of the most important considerations. The Mini-Cog may have such advantages as a cognitive screening tool that is receiving increasing attention (Borson et al., 2006; Tsoi et al., 2017). The Mini-Cog is a concise cognitive assessment tool. It has been validated and takes only a few minutes to complete (Tam et al., 2018).

In this study, we used the Mini-Cog test for preoperative cognition screening and hypothesized that PCI screened by Mini-Cog is highly associated with POD.

## 2. Methods

### 2.1. Study population

This single-center prospective observational cohort study was performed in the People’s Hospital of Henan Province, China. The study was approved by the Ethics Review Committee of Henan Provincial People’s Hospital (2020-99) and was also registered in ClinicalTrials.gov (NCT05798767). Elderly patients who were hospitalized between February 2022 and July 2022 and required elective thoracic surgery were recruited into the study. Informed consent was obtained from all subjects involved in the study. We performed preoperative screening of potential subjects the day before surgery. Inclusion criteria were (1) at least 65 years old, (2) elective thoracic surgery, and (3) under general anesthesia. We excluded patients with (1) a history of psychiatric disorders or use of any antipsychotic drugs, (2) preoperative diagnosis of dementia, (3) ASA score > 3, (4) history of overt stroke or brain tumor, (5) severe visual, hearing, or physical dysfunction unable to complete the scale, (6) advanced malignant tumors of the chest with distant metastases to bone, liver, etc., and (7) history of general anesthesia surgery in the last 6 months. All patients signed the informed consent. All patients signed the informed consent.

### 2.2. Preoperative screening

The day before surgery, we collected data points on demographics and hospital episodes through the electronic medical record. Mini-Cog and the Mini-mental State Examination (MMSE) were used for screening cognitive impairment. The Mini-Cog consists of word memory (3 points, 1 point per word) and a clock drawing test (2 points, 1 point for drawing the numbers and 2 points for drawing the numbers and hands). The total score was 5. We considered a Mini-Cog score of 3 or less as cognitive dysfunction (Buysse et al., 1989). The MMSE consists of 30 short questions, with a total score of 30. An MMSE score of less than 27 is considered cognitive dysfunction. The time spent on Mini-Cog test and MMSE assessment were recorded.

Depression screening using Patient Health Questionnaire-9 (PHQ-9), and sleep quality assessment using Pittsburgh Sleep Quality Index (PSQI) were also performed. All tests were performed in a quiet environment to exclude environmental disturbances. Preoperative assessments were performed by senior anesthesiologists. The investigators received training from specialized psychiatrists.

### 2.3. Perioperative management

The patients followed the usual anesthetic protocol in our institute and were not premedicated. Intraoperative monitoring of patients included electrocardiogram, non-invasive blood pressure, oxygen saturation, end-tidal partial pressure of carbon dioxide, end-tidal concentration of sevoflurane, temperature, and urine output. Invasive blood pressure and central venous pressure were measured if needed. General anesthesia was intravenously induced with propofol 1–2 mg/kg, sufentanil 0.3–0.5 μg/kg, and cis-atracurium (0.15–0.2 mg/kg) or rocuronium (0.6–0.9 mg/kg). Intraoperatively, intravenous and inhalation anesthetics were used to maintain the anesthetic state. Intravenous pump infusion of propofol was 4–12 mg/kg/h. Remifentanil was pumped at a maintenance rate of 0.12–1.8 μg/kg/min. Continuous pumping of cisatracurium 1–2 μg/kg/min (0.06–0.12 ml/kg/h) was started 30 min after induction of anesthesia, with the possibility of additional injections as needed. Inhalation anesthetics were administered using sevoflurane with a minimum alveolar concentration (MAC) of 0.8–1. BIS values were maintained at 40–60. Propacetamol 2 g and tropisetron 5 mg were given before surgery ended. Anesthesiologists were blinded to the results of patients’ preoperative evaluations. Multi-modal analgesia was used for patients. Patients should be controlled as much as possible to have an NRS score of ≤4 on the Numeric Rating Scale for Pain at Rest after surgery, and opioids or NSAIDS may be used. If the patient’s NRS score at rest is greater than 4 at the postoperative follow-up, pharmacological interventions should be performed.

### 2.4. Postoperative follow-up

After surgery, follow-up visits were performed by an investigator on days 1–5. The investigator specializing in follow-up were unknown of patients’ preoperative evaluations. Delirium evaluation was conducted twice daily using The Short Confusion Assessment Method (CAM) at 8–10 and 15–17 points. The CAM scale and a review of the postoperative history (electronic medical record, physician and family interviews) were used to determine if the patient was in POD. If POD was determined, the patient and his history were reviewed by other investigators within 3 days to ensure consistency of the POD assessment. The pain assessment was also completed simultaneously, and postoperative pain was defined for NRS score ≥4. We recorded postoperative pulmonary-related complications (atelectasis, pneumonia, respiratory failure within 7 days postoperatively), postoperative cardiovascular complications (postoperative arrhythmias, myocardial infarction, cerebral infarction, etc.), and other complications (sepsis, blood transfusion, etc.). In addition, the length of hospital stay was recorded. After 6 months, all patients received telephone follow-ups.

### 2.5. Statistical analysis

According to the references and the results of our pretest, the incidence of PCI in elderly patients was 40%, the probability of POD in elderly patients with normal preoperative cognition was 20%, and the probability of POD in elderly patients in the presence of PCI was 46%. With a test efficacy (1-alpha) of 80%, a *P*-value of 0.05, and a postoperative shedding rate of 10%, 150 patients were calculated to be included. The sample size of this study was calculated using PASS 15.0 software.

We first evaluated the data for outliers and performed multiple interpolations for missing data. Normally distributed continuous variables were depicted as mean ± standard and were analyzed by *t*-test. Non-normally distributed continuous variables were presented as median and interquartile range (IQR) and analyzed by using Mann–Whitney U test. We used counts and rates (%) to describe qualitative variables and used the Chi-square test or Fisher’s exact test to analyze. Comparisons of variables between groups were performed using the tableone R package. The association between perioperative variables and delirium was identified by univariate and multifactorial logistic regression analysis using the rms R package. In the univariate analysis, the covariates of age, history of alcohol consumption, PCI, PHQ-9 score, ASA grade, duration of anesthesia, Artificial airways, and Intraoperative fluid volume, which had *p*-values < 0.2 were entered into a stepwise regression model. The multifactor logistic regression model was eventually developed. The prediction model was validated by using the Bootstrap method and calculating the area under the ROC. The comparison of the time spent on MMSE and the Mini-Cog was analyzed by the paired-sample *t*-test. Using the MMSE as criteria for diagnosing PCI, we plotted the receiver operating characteristic curve (ROC) for the Mini-Cog. The ROC curves are plotted using the pROC package. And other drawings are done using the ggplot2 R package. *P* < 0.05 is considered to be statistically significant. R version 3.1.4 was used to perform all data analyses (R Development Core Team, Vienna, Austria).

## 3. Results

### 3.1. Characteristics of patients

**FIGURE 1:**
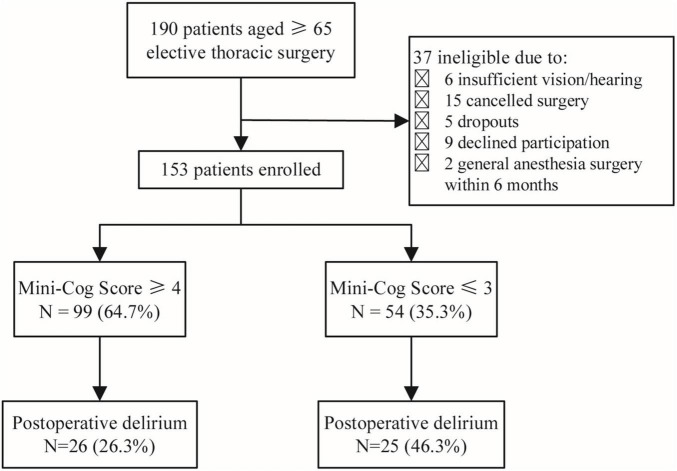
Flow diagram.

One hundred and ninety elderly patients aged ≥65 years planning to undergo elective thoracic surgery were recruited for this project. There were 37 patients excluded, including 6 patients who were unable to complete the screening due to visual or hearing impairment, 15 patients whose surgery was canceled, 5 who dropped out, 9 who refused to participate in the study, and 2 who had a history of general anesthesia surgery within 6 months. Finally, 153 participants were included in the research (Figure 1).

According to Mini-Cog scores, participants were divided into two groups: 99 patients with a Mini-Cog score of 3 or 4 were in the normal group, and patients with a Mini-Cog score of 3 or less were in the PCI group. Residence (rural or urban), education level, and hypertension were significantly different between the two groups (*P* < 0.05). The PCI group had a lower education level and higher hypertension prevalence and were more likely to live in rural areas (Table 1).

**TABLE 1:** Characteristics of participants between the PCI group and the normal group according to Mini-Cog scores.

Variables	Total (*N* = 153)	Mini-Cog ≤ 3 (*N* = 54)	Mini-Cog ≥ 4 (*N* = 99)	*P*-value
Female	66 (43.1%)	28 (51.9%)	38 (38.4%)	0.151
Age, years old	70 [67;73]	70 [68;73]	69 [67;73]	0.509
Place of residence				<0.001
Urban	73 (47.7%)	11 (20.4%)	62 (62.6%)	
Rural	80 (52.3%)	43 (79.6%)	37 (37.4%)	
Education level				<0.001
College degree	15 (9.8%)	2 (3.7%)	13 (13.1%)	
High school diploma	64 (41.8%)	11 (20.4%)	53 (53.5%)	
Less than high school	74 (48.4%)	41 (75.9%)	33 (33.3%)	
Smoking (Yes, n, %)	62 (40.5%)	16 (29.6%)	46 (46.5%)	0.064
Alcohol consumption (Yes, n, %)	40 (26.1%)	13 (24.1%)	27 (27.3%)	0.812
Marital status				0.098
Divorced or widowed	11 (7.19%)	1 (1.85%)	10 (10.1%)	
Married	142 (92.8%)	53 (98.1%)	89 (89.9%)	
Body mass index, kg/m^2^	24.2 (3.4)	23.7 (3.4)	24.4 (3.4)	0.230
History of general anesthesia	66 (43.1%)	19 (35.2%)	47 (47.5%)	0.195
Comorbidities				
Hypertension	60 (39.2%)	28 (51.9%)	32 (32.3%)	0.028
Diabetes	27 (17.6%)	12 (22.2%)	15 (15.2%)	0.382
Coronary heart disease	25 (16.3%)	10 (18.5%)	15 (15.2%)	0.757
Cerebrovascular disease	14 (9.2%)	8 (14.8%)	6 (6.1%)	0.085
CCI	0.9 (1.0)	1.0 (1.1)	0.8 (0.9)	0.261
Preoperative evaluation				
Mini-Cog score	4.0 (1.0)	2.7 (0.5)	4.7 (0.5)	<0.001
MMSE score	26.4 (6.1)	23.8 (9.6)	27.8 (1.6)	0.004
PHQ-9 score	1 [1;2]	1 [1;2]	1 [1;2]	0.422
PSQI score	7 [5;9]	7 [5;10]	7 [5;9]	0.554
ASA score				1.000
II	102 (66.7%)	36 (66.7%)	66 (66.7%)	
III	51 (33.3%)	18 (33.3%)	33 (33.3%)	
Surgery method				1.000
Thoracotomy	6 (3.9%)	2 (3.7%)	4 (4.0%)	
Thoracoscopic surgery	147 (96.1%)	52 (96.3%)	95 (96.0%)	
Duration of anesthesia, min	190 [135;250]	205 [154;255]	180 [120;243]	0.103
Intraoperative fluid volume, ml	1,305 (600)	1,390 (650)	1,259 (569)	0.218
Postoperative complications				
Postoperative delirium	51 (33.3%)	25 (46.3%)	26 (26.3%)	0.020
Atelectasis	17 (11.1%)	7 (13.0%)	10 (10.1%)	0.788
Respiratory failure	4 (2.6%)	2 (3.7%)	2 (2.0%)	0.614
Pulmonary infection	19 (12.4%)	8 (14.8%)	11 (11.1%)	0.684
Blood transfusion	18 (11.8%)	7 (13.0%)	11 (11.1%)	0.938
Atrial fibrillation	2 (1.3%)	2 (3.7%)	0 (0.0%)	0.123
Postoperative pain (NRS ≥ 4)	49 (32.0%)	15 (27.8%)	34 (34.3%)	0.515
Length of hospital stay, days	7 [5;11]	7 [4;10]	7 [5;11]	0.709

MMSE, the Mini-mental State Examination; PHQ-9, Patient Health Questionnaire-9; PSQI, Pittsburgh Sleep Quality Index; ASA, American Society of Anesthesiologist; NRS, Numeric Rating Scale.

A total of 51 patients (33.3%) developed POD. Patients in the PCI group were more likely to develop POD compared to the Normal group (26.3 vs. 46.3%, *P* = 0.020) (Table 1).

### 3.2. Mini-Cog scores of patients

**FIGURE 2:**
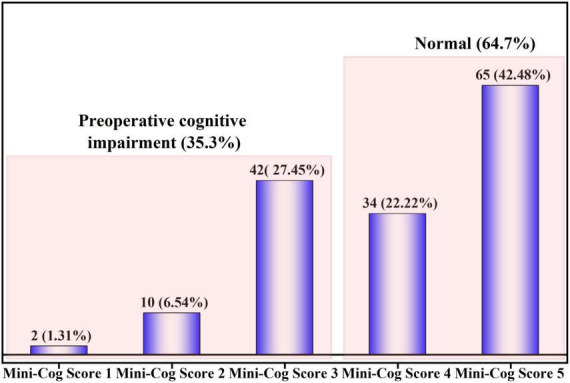
Preoperative cognitive impairment in elderly thoracic surgery patients.

A total of 54 (35.3%) had PCI as identified by the Mini-Cog score. The Mini-Cog score distribution of the patients is shown in Figure 2.

**FIGURE 3:**
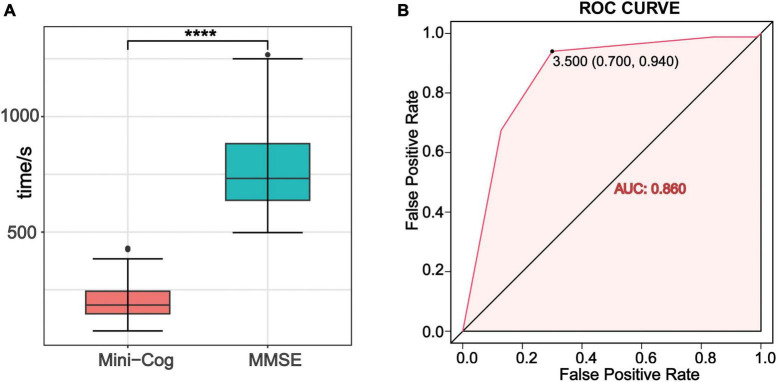
**(A)** Comparison of the time spent on MMSE and the Mini-Cog test. ****P ≤0.0001. **(B)** The receiver operating characteristic curve (ROC) of Mini-Cog for discriminate delirium. AUC was 0.860. The optimal diagnostic threshold for Mini-Cog was 3.5, with sensitivity of 0.94 and specificity of 0.70.

The average time to assess cognition on the MMSE was 764 ± 157 s, and the average time to complete the Mini-Cog was 200 ± 71.4 s. Mini-Cog took much less time than MMSE (*P* < 0.001) (Figure 3A). The ROC of the Mini-Cog was plotted using the MMSE as a criterion for diagnosing PCI. The area under the curve (AUC) was 0.860, with an optimal diagnostic threshold of 3.5 (Figure 3B).

### 3.3. POD prediction model

In the multivariable analysis, PCI (OR = 2.37; 95% confidence interval [CI], 1.1∼5.18; *P* = 0.028), advanced age (OR = 1.13; 95% CI, 1.03∼1.24; *P* = 0.009), ASA grade III (OR = 2.75; 95% CI, 1.25∼6.13; *P* = 0.012), and longer duration of anesthesia (OR = 1.01; 95% CI, 1∼1.01; *P* = 0.007) were related to POD (Table 2).

**TABLE 2:** Association of perioperative variables with delirium identified by univariate and multi-factorial regression analysis.

Variables	Univariate analysis		Multivariate analysis	
	*P*. overall	OR (95% CI)	OR (95% CI)	*P*-value
Age, years old	0.001	1.14 (1.05∼1.24)	1.13 (1.03∼1.24)	0.009
Alcohol consumption	0.044	2.29 (1.09∼4.84)		
PCI:	0.020	2.42 (1.21∼4.89)	2.37 (1.10∼5.18)	0.028
Mini-Cog score ≥ 4				
Mini-Cog score ≤ 3				
PHQ-9 score	0.105	1.06 (0.89∼1.25)		
ASA score	0.002		2.75 (1.25∼6.13)	0.012
II				
III				
Duration of anesthesia, min	0.008	1.01 (1.00∼1.01)	1.01 (1.00∼1.01)	0.007
Artificial airways	0.080			
Single-lumen endobronchial tube		3.17 (1.24∼8.37)		
Laryngeal mask airway		1.22 (0.06∼13.30)		
Double-lumen right bronchus tube		1.01 (0.44∼2.26)		
Double-lumen left bronchus tube				
Intraoperative fluid volume, ml	0.055	1.00 (1.00∼1.00)		

PCI, Preoperative Cognitive Impairment; PHQ-9, Patient Health Questionnaire-9; ASA, American Society of Anesthesiologists; OR, odds ratio; CI, confidence interval.

**FIGURE 4:**
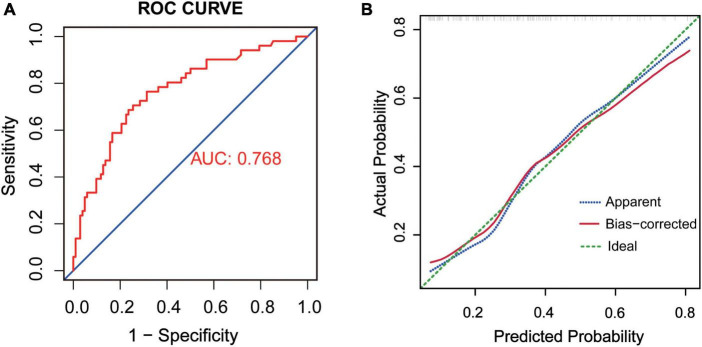
**(A)** ROC of the prediction model, AUC = 0.768. **(B)** Internal validation by Bootstrap resampling method. The X-axis was the likelihood of the outcome predicted by the model and the Y-axis was the value obtained from the actual observation. Bias-corrected was the corrected curve, while diagonal Ideal was the ideal curve.

The ROC of this delirium prediction model was plotted (Figure 4A). AUC was 0.768, with a 95% CI of 0.688–0.850. Therefore, the model had good discrimination. Also, we used the Bootstrap resampling method to validate the logistic regression model. The calibration curve was similar to the ideal curve, so the model had a good calibration ability (Figure 4B).

**FIGURE 5:**
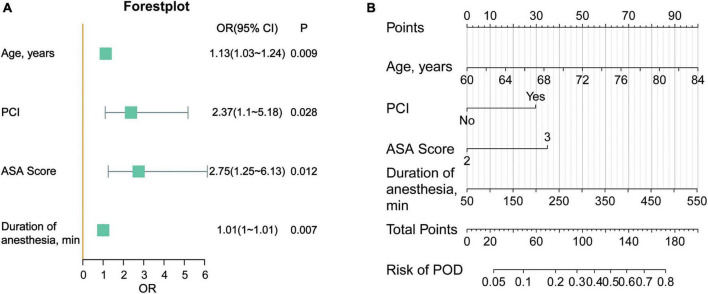
**(A)** Forestplot of the delirium prediction model. **(B)** Nomogram of the prediction model for POD. The score of each variable is obtained according to the scale above the variable, and the total score is calculated by adding the scores of each variable. Then from the total score downward, we can get the predicted probability of the patient.

Furthermore, we visualized the model with a forest plot and a nomogram to facilitate rapid clinical judgment (Figures 5A, B).

### 3.4. Outcomes of patients with POD

Further analysis was performed on elderly patients who developed POD. POD increased the risk of postoperative pulmonary atelectasis (6.86 vs. 19.6%, *P* = 0.036) and pulmonary infection (7.84 vs. 21.6%, *P* = 0.030), increased the incidence of un-planned ICU admission (8.82 vs. 25.5%, *P* = 0.012), and prolonged the length of postoperative hospital stay (7 vs. 8, *P* = 0.029). There were three deaths in the delirium group at 6 months follow-up and zero deaths in the non-delirium group (0.00 vs. 5.88%, *P* = 0.036) (Table 3).

**TABLE 3:** Postoperative outcomes in patients with and without delirium.

Variables	POD		*P*-value
	No = 102	Yes = 51	
Blood transfusion	8 (7.84%)	10 (19.6%)	0.062
Atelectasis	7 (6.86%)	10 (19.6%)	0.036
Respiratory failure	2 (1.96%)	2 (3.92%)	0.601
Pulmonary embolism	1 (0.98%)	0 (0.00%)	1.000
Pneumothorax	4 (3.92%)	4 (7.84%)	0.442
Pleural effusion	2 (1.96%)	3 (5.88%)	0.334
Pulmonary infection	8 (7.84%)	11 (21.6%)	0.030
Incisional infection	1 (0.98%)	1 (1.96%)	1.000
Sepsis	0 (0.00%)	1 (1.96%)	0.333
Postoperative atrial fibrillation	1 (0.98%)	1 (1.96%)	1.000
Postoperative ICU admission	9 (8.82%)	13 (25.5%)	0.012
Length of ICU stay, days	1.44 (1.33)	1.85 (1.82)	0.557
Length of hospital stay, days	7 [4;10]	8 [5;12]	0.029
Mortality after 6 months	0 (0.00%)	3 (5.88%)	0.036

POD, postoperative delirium; ICU, intensive care unit.

## 4. Discussion

This study demonstrated that PCI increases the risk of POD, and Mini-Cog is an appropriate tool to screen for PCI. We also developed and validated a delirium prediction model for elderly surgical patients.

Preoperative cognitive function screening is often ignored because of time and resource restrictions. In this study, the prevalence of preoperative cognitive dysfunction was 35.3% in older patients, which was consistent with most previous studies. The survey by Culley et al. (2016), utilized Mini-Cog cognitive screening tool and found that 25–33% of geriatric patients planning elective surgery may have PCI. A Meta-analysis by Kapoor et al. (2022), indicated that the prevalence of cognitive impairment in elective non-cardiac surgery was 37.0%, and it was 50.0% in emergency surgery.

Educational attainment has a significant effect on cognitive function. Patients with good education had a lower probability of cognitive impairment. People with better education tend to be more subjective and more adaptable to learning knowledge (Clouston et al., 2012). This may stimulate brain cells, resulting in structural and functional adaptations in the brain. The connections between neuronal synapses are enhanced, and the brain has sufficient neuronal reserves. This makes the brain tolerant to several structural or functional brain cell deficits and delays the progression of senile brain atrophy (Cabeza et al., 2018; Lövdén et al., 2020). Recently, the cognitive reserve has been proposed as an emerging dynamic concept that is facilitated by educational attainment and physical activity (Kassie et al., 2017). Saleh et al. (2015), suggested that patients with adequate preoperative cognitive reserve have a lower risk of postoperative cognitive dysfunction.

Older patients who reside in rural areas have a higher prevalence of cognitive dysfunction. On the one hand, pastoral older adults have lower educational attainment, which affects their cognitive abilities; on the other hand, because of medical resource limitations, rural health care and nursing conditions are worse, making it difficult to meet the demands of older patients with cognitive dysfunction. It has been identified hypertension is strongly associated with cognitive impairment (Ungvari et al., 2021; Antonazzo et al., 2022). A cross-sectional study compared cognitive function in 221 (71 normotensive and 150 hypertensives) patients. The results showed that hypertension probably causes cognitive decline (Muela et al., 2017). Recent evidence suggests that hypertension may change the structure and function of brain through cerebral vascular remodeling processes. It may interrupt brain autoregulation and reduce cerebral perfusion, thereby increasing the risk of cognitive dysfunction (Walker et al., 2017).

Cognitive impairment as an evolving concept and clinical diagnostic entity has spawned a variety of cognitive assessment criteria for the screening of cognitive impairment. MMSE is widely used for dementia detection and is also applied for detecting mild cognitive impairment (Tsoi et al., 2015). It has been suggested that the Mini-Cog has a stronger ability to identify cognitive impairment than the MMSE (Borson et al., 2000). Further research by Borson et al. (2005), has shown that Mini-Cog has higher overall accuracy than the MMSE (83 and 81%) in distinguishing mild cognitive impairment from normal cognition and is less affected by low education levels. In our study, the MMSE was considered a criterion for diagnosing PCI. The Mini-Cog has a high accuracy with an area under the ROC of 0.86. Moreover, the average time spent on MMSE was 764 ± 157 s, and the average time to complete the Mini-Cog test was 200 ± 71.4 s. Using the Mini-Cog for preoperative cognitive assessment is simpler, faster, and more acceptable for patients and healthcare professionals, which makes it possible to perform cognitive screening preoperatively routinely.

Numerous studies support that the elderly with PCI is more likely to develop delirium postoperatively (Culley et al., 2017; Susano et al., 2020; Tiwary et al., 2021; Yajima et al., 2022). A recent large retrospective cohort study showed that one-fifth of patients aged ≥70 years undergoing elective surgery had PCI. And patients in the presence of PCI are more likely to develop POD (OR = 3.3; 95% CI, 2.3–4.7) (Weiss et al., 2022). Patients with cognitive impairment have reduced cortical function, reduced number of neuronal cells, and reduced blood supply. The activity of acetylcholinesterase and carbonic anhydrase is diminished, and the number of muscarinic receptors and 5-hydroxytryptamine receptors is decreased. With cholinergic neurodegeneration in the brain, acetylcholine and presynaptic choline receptors are reduced, which makes it more likely to lead to delirium (Egberts et al., 2021).

The study investigated the predictive value of Mini-Cog for postoperative delirium in elderly patients by addressing critical clinical issues and came up with reliable conclusions. It is instructive and innovative for clinical practice. However, there are a few limitations of this study. (1) Mini-Cog scale has a high sensitivity but relatively low specificity for identifying cognitive impairment. There may be false positives in our screening for PCI using Mini-Cog. (2) Due to the fluctuating nature of delirium, we may have missed the occurrence of delirium during follow-up. The actual incidence of delirium may be higher. (3) Although we used multifactorial regression analysis to minimize the effect of confounding factors. However, there may still be potential confounders not included in the analysis, causing bias. (4) This study was a single-center study of elderly patients undergoing thoracic surgery. Multicenter studies with large samples are still needed to investigate further the relationship between PCI screened by Mini-Cog with POD in elderly patients with various types of surgery.

## 5. Conclusion

Preoperative cognitive impairment is prevalent in elderly surgical patients and it increases the risk of POD. There is no consensus on a cognitive screening tool for the preoperative setting. The Mini-Cog is a brief and valid cognitive assessment scale and takes much less time than MMSE. It is appropriate for preoperative cognitive screening. Screening for PCI offers the possibility of providing personalized care to geriatric thoracic patients so that adverse postoperative outcomes can be mitigated.

## Data availability statement

The original contributions presented in this study are included in this article/supplementary material, further inquiries can be directed to the corresponding author.

## Ethics statement

The studies involving human participants were reviewed and approved by the Ethics Review Committee of Henan Provincial People’s Hospital (2020-99). The patients/participants provided their written informed consent to participate in this study. Written informed consent was obtained from the individual(s) for the publication of any potentially identifiable images or data included in this article.

## Author contributions

FL: methodology, data collection, data analysis, and writing original draft preparation. MM: data analysis. NL: patient recruitment. JZ: investigation. MS: data curation. JQZ: supervision, project administration, and manuscript revision. All authors reviewed and approved the manuscript.
